# Analysis of the correlation between core competencies of pediatric nurses and occupational benefit perception and its influencing factors

**DOI:** 10.3389/fped.2025.1559572

**Published:** 2025-05-26

**Authors:** Huihui Zhang, Jianxin Wang, Chunhui Li, Xinyu Liu, Qianmei Xiang

**Affiliations:** ^1^ Department of Operating Room, Tangshan Maternal and Child Health Hospital, Tangshan, Hebei, China; ^2^ Department of Pediatric Surgery, Tangshan Maternal and Child Health Hospital, Tangshan, Hebei, China; ^3^ Department of Pediatrics, Tangshan Maternal and Child Health Hospital, Tangshan, Hebei, China

**Keywords:** pediatric nurse specialists, core competencies, career benefits perception, correlation, standardized training

## Abstract

**Purpose:**

Analyzed the correlation between core competencies and career benefits in pediatric nurse specialists, and examined the factors influencing core competencies in pediatric nurse specialists. Provided references for pediatric nursing managers to improve nurses’ core competencies and stabilize the standardized training of pediatric nurse specialists.

**Method:**

Using a convenience sampling method, pediatric nurse specialists from Hebei Children's Hospital, Qinhuangdao Maternal and Child Health Hospital, and Baoding Children's Hospital were selected as study subjects between August and September 2023. A questionnaire survey was conducted using a general information questionnaire, a career benefit scale, and a specialist pediatric nurse core competency scale. Pearson correlation and multiple linear regression analyses were employed to examine relationships and influencing factors.

**Results:**

The total score of career benefits perception for the 128 pediatric nurse specialists was 117.72 ± 12.34, and the total score of core competencies was 132.23 ± 18.96, both of which were above-average. There were significant differences in the total core competency scores among pediatric nurse specialists of different ages, years of work experience, technical titles, and employment forms. There was a significant positive correlation between the core competencies of pediatric nurse specialists and each dimension of career benefits perception (*p* < 0.01). Multiple linear regression analysis showed that employment form, years of work experience, and total score of career benefits perception were independent influencing factors of the core competencies of pediatric nurse specialists.

**Conclusion:**

The career benefits perception and core competencies of pediatric nurse specialists are both at an above-average level, with a close and mutually influential relationship. The form of employment, years of experience, and total score of career benefits influence the core competencies of pediatric nurse specialists. Nursing managers should take targeted intervention measures based on these influencing factors to improve the core competencies of pediatric nurse specialists.

## 1 Introduction

Specialist nurseshave received specific training in a particular field or specialty, possessing advanced professional knowledge and skills to provide high-level nursing services (1, 2). Compared to general registered nurses, specialist nurses undergo training and examinations for the specialized skills and knowledge required in areas such as emergency care, surgery, mental health, and pediatrics. They have deeper expertise and experience, enabling them to more precisely meet and address patient needs, thereby promoting recovery and health management (3, 4). Among them, pediatric nurse specialists play a crucial role in child care. Their pediatric nursing knowledge and skills enable them to provide personalized care and health education to children and their families, coordinate medical teams, handle emergencies, and offer emotional support. This promotes children's healthy development and recovery, making them an indispensable part of pediatric nursing (5–7).

Nurse core competencies refer to the essential skills, knowledge, and attributes that nurses must possess in clinical practice, as defined by Chen et al. (8). This framework comprises six dimensions validated in pediatric nursing contexts: (1) clinical judgment and decision-making, (2) specialized pediatric knowledge, (3) technical proficiency, (4) interdisciplinary communication and collaboration, (5) leadership and management, and (6) commitment to professional development (8). These dimensions align with the International Council of Nurses' competency standards for advanced practice nurses (9), ensuring a holistic evaluation of pediatric nursing expertise. These core competencies cover a wide range of areas, including but not limited to clinical technical operations, patient care, communication skills, teamwork, problem-solving abilities, professional development, and continuous learning (1, 7). Developing nurse core competencies ensures that nurses can competently perform various nursing roles, provide safe, effective, and high-quality nursing services, and continuously adapt to the ever-changing healthcare environment and patient needs (7). The concept of core competence was first proposed by foreign scholars Prahalad and Hamel in 1990. In 2003, Chinese scholars introduced the study of core competencies into the nursing field (10).

Pediatric nurse specialists play a crucial role in clinical practice, and the importance of enhancing their core competencies cannot be overstated (11). The unique characteristics of pediatric patients require nurses to possess a higher level of professional knowledge and skills to address various pediatric diseases and care needs (12). Additionally, the complexity and challenges of pediatric nursing demand a solid professional foundation and the ability to respond effectively (13). Moreover, continuously improving the core competencies of pediatric nurse specialists helps enhance the quality and standards of pediatric nursing services, providing safer and more effective care for pediatric patients and promoting their physical and mental health (14, 15). Therefore, strengthening the training and education of pediatric nurse specialists and improving their core competencies is of great significance for optimizing the pediatric nursing system and improving the quality of pediatric medical services.

In recent years, the relationship between nurses' career benefits perception and their core competencies has garnered much attention from scholars. This study analyzed the current status of pediatric nurse specialists' career benefits perception and core competencies, explores the relationship between core competencies and career benefits perception, and examines other influencing factors of nurses' core competencies, providing references for pediatric nursing managers to improve nurses' core competencies.

## 2 Materials and methods

### 2.1 Study subjects

From August to September 2023, pediatric nurse specialists from Hebei Children's Hospital, Qinhuangdao Maternal and Child Health Hospital, and Baoding Children's Hospital were selected for the survey. This study was conducted by the principles outlined in the Declaration of Helsinki. Ethical approval was obtained from the Ethics Committee of Tangshan Maternal and Children's Health Hospital. Inclusion criteria: (1) Registered nurses with ≥1 year of experience working in pediatric specialties; (2) Nurses who have undergone specialist training and have pediatric specialist nurse qualifications; (3) Nurses engaged in pediatric clinical care; (4) Nurses who are informed and willing to cooperate with the survey. Exclusion criteria: (1) Nurses in pediatric standardized training, rotation, or advanced study; (2) General ward nurses temporarily assigned to or supporting pediatric departments; (3) Nurses not on duty during the survey period; (4) Full-time office nurses and those not directly involved in pediatric clinical care.

### 2.2 Research tools

#### 2.2.1 General information questionnaire

Data were collected via the Wenjuanxing platform between August and September 2023. To ensure validity, two researchers independently cross-checked entries against raw questionnaires. Missing data (<5%) were addressed using mean imputation after confirming missing-at-random assumptions via Little's MCAR test. Developed based on relevant literature (16, 17) and the actual clinical work of nurses, this questionnaire includes information on gender, age, education level, marital status, professional title, employment form, and years of work experience.

#### 2.2.2 Pediatric specialist nurse core competency scale

The Pediatric Specialist Nurse Core Competency Scale, developed by the primary researcher, includes five dimensions: “Basic Professional Qualities” (4 items), “Clinical Nursing Practice Ability” (11 items), “Management and Guidance Ability” (6 items), “Communication and Coordination Ability” (4 items), and “Professional Development Ability” (9 items), totaling 34 items. Each item is rated on a Likert 5-point scale, with 1 = “Very Inapplicable”, 2 = “Inapplicable”, 3 = “Sometimes Applicable”, 4 = “Applicable”, and 5 = “Very Applicable”. The total score ranges from 34 to 170 points, with higher scores indicating higher levels of core competency in pediatric nurse specialists. The scale's overall Cronbach's *α* coefficient is 0.967, with Cronbach's *α* coefficients for each dimension ranging from 0.883 to 0.953. The split-half reliability of the scale is 0.861, with split-half reliability for each dimension ranging from 0.767 to 0.918. The test-retest reliability of the scale is 0.985, with test-retest reliability for each dimension ranging from 0.719 to 0.919.

#### 2.2.3 Career benefits perception scale

The “Nurse Career Benefits Perception Questionnaire”, developed by Hu Jing et al. (9), is used. The questionnaire includes five dimensions: “Positive Occupational Perception” (5 items), “Good Nurse-Patient Relationship” (6 items), “Family and Friends Recognition” (7 items), “Team Belongingness” (5 items), and “Personal Growth” (6 items), totaling 29 items. Each item is rated on a Likert 5-point scale, with 1 = “Very Dissatisfied”, 2 = “Dissatisfied”, 3 = “Sometimes Satisfied”, 4 = “Satisfied”, and 5 = “Very Satisfied”. The total score ranges from 29 to 145 points, with higher scores indicating stronger career benefits perception in pediatric nurse specialists. The overall Cronbach's *α* coefficient for the questionnaire is 0.958, with Cronbach's *α* coefficients for each dimension ranging from 0.821 to 0.893.

#### 2.2.4 Survey method

The survey was conducted using an online questionnaire distribution method. The questionnaire content was imported into Wenjuanxing to create an online questionnaire, and the webpage link was retained. After obtaining consent and support from the nursing departments and pediatric nursing supervisors of each hospital, the purpose of the survey, the filling method, and precautions were explained. The pediatric nursing supervisors distributed the survey questionnaire link to pediatric nurse specialists via WeChat groups for completion. The questionnaire was completed anonymously and voluntarily, and each IP address was allowed to fill out the questionnaire only once. After exporting the data, an Excel database was established, and data entry was cross-checked by two individuals to ensure the accuracy and authenticity of the survey data.

### 2.3 Statistical methods

The data were entered using the double-entry method and analyzed using SPSS 24.0 statistical software, with normality assessed via Shapiro–Wilk tests and homogeneity of variance via Levene's test. Descriptive statistics (percentages, means, standard deviations, medians, quartiles) summarized the data, while between-group comparisons employed independent t-tests (two groups) or one-way ANOVA (multiple groups) for normally distributed data (with effect sizes reported as Cohen's d or partial eta-squared) and Mann–Whitney U or Kruskal–Wallis H tests (with effect sizes as r or epsilon-squared) for non-normal data, using Bonferroni-corrected *post-hoc* tests where applicable. Multiple linear regression assumptions (normality, homoscedasticity, multicollinearity) were verified through residual diagnostics (Q-Q plots, Breusch-Pagan test, VIF < 2.0), with adjusted R² reported for model fit; all analyses used *p* < 0.05 for significance with 95% CIs where appropriate.

## 3 Results

### 3.1 General information of pediatric nurse specialists

The sample size was determined using G*Power 3.1, with an effect size of 0.3, α = 0.05, and power = 0.8, yielding a minimum requirement of 98 participants (18). A total of 134 questionnaires were distributed in this survey, with 128 valid questionnaires collected, resulting in a response rate of 95.52%. Multiple linear regression assumptions were verified, including normality (Shapiro–Wilk *p* > 0.05), homoscedasticity (Breusch-Pagan *p* = 0.21), and absence of multicollinearity (VIF < 2.0). The general information of the 128 specialist nurses included in the study was analyzed and organized. The pediatric specialist nurse group was highly feminized, with females accounting for 98.44% (126 individuals) and males accounting for only 1.56% (2 individuals). The age structure is mainly concentrated in the 21–30 age group, accounting for 64.84% (83 individuals), followed by the 31–40 age group, accounting for 28.91% (35 individuals), while those aged over 40 account for 6.25% (8 individuals). Regarding marital status, married nurses account for 76.56% (98 individuals), unmarried individuals account for 21.88% (28 individuals), and divorced individuals account for 1.56% (2 individuals). The majority of nurses have 6–10 years of work experience, accounting for 47.66% (61 individuals). Those with ≤5 years of experience account for 21.88% (28 individuals), 11–20 years account for 23.44% (30 individuals), and >20 years account for 7.03% (9 individuals). In terms of educational qualifications, 85.16% (109 individuals) have a bachelor's degree, and 14.84% (19 individuals) have a master's degree or above. Regarding technical titles, nurse account for 59.38% (76 individuals), supervisor nurse account for 32.03% (41 individuals), and associate chief nurse account for 8.59% (11 individuals). In terms of employment forms, 73.44% (94 individuals) are on long-term contracts, 17.97% (23 individuals) are on temporary contracts, and 8.59% (11 individuals) are on permanent contracts. Overall, these data reflect that the majority of pediatric nurse specialists in this study were female, highly educated, and had varying levels of clinical experience, primarily employed on long-term contracts (Table 1**)**.

**Table 1:** General information of pediatric specialist nurses.

Information		Number (*n*)	Percentage (%)
Sex	Male	2	1.56
	Female	126	98.44
Age (years)	21–30	83	64.84
	31–40	37	28.91
	>40	8	6.25
Marital status	Unmarried	28	21.88
	Married	98	76.56
	Divorced	2	1.56
Years of work (years)	≤5	28	21.88
	6–10	61	47.66
	11–20	30	23.44
	>20	9	7.03
Academic qualifications	Bachelor's Degree	109	85.16
	Master and above	19	14.84
Technical title	Nurse	76	59.38
	Supervisor nurse	41	32.03
	Associate chief nurse	11	8.59
Employment form	Temporary contract	23	17.97
	Long-term contract	94	73.44
	Career	11	8.59

### 3.2 Core competency scores of pediatric nurse specialists

Pediatric nurse specialists have demonstrated high levels of core competency in basic professional qualities, clinical nursing practice ability, management and guidance ability, communication and coordination ability, and professional development ability. Among these, the score for clinical nursing practice ability is the highest (47.86 ± 5.93), while the score for management and guidance ability is relatively lower (19.08 ± 3.19), indicating that nurses excel in practical nursing operations but still have room for improvement in management and guidance. Overall, the total score for nurses' core competency is (132.23 ± 18.96), indicating strong overall abilities among nurses but with individual differences (Table 2).

**Table 2:** Core competency scores of pediatric specialist nurses [(mean ± SD), M(Q1, Q3), *n* = 128].

Dimension	Items	Score range	Score
Basic Professional Qualities	4	4–20	17.00 (15.00, 18.50)
Clinical Nursing Practice Ability	11	11–55	47.86 ± 5.93
Management and Guidance Ability	6	6–30	19.08 ± 3.19
Communication and Coordination Ability	4	4–20	16.00 (15.00, 18.00)
Professional Development Ability	9	9–45	32.03 ± 5.53
Total Score	34	34–170	132.23 ± 18.96

### 3.3 Career benefits perception scores of pediatric nurse specialists

The total score for the career benefits perception of pediatric nurse specialists is (117.72 ± 12.34). The scores for each dimension, including positive occupational perception, good nurse-patient relationship, family and friends' recognition, team belongingness, and personal growth, are detailed in Table 3.

**Table 3:** Career benefits perception scores of pediatric specialist nurses [(mean ± SD), M(Q1, Q3), *n* = 128].

Dimension	Items	Score Range	Score
Positive Occupational Perception	5	5–25	20.00 (19.00, 22.00)
Good Nurse-Patient Relationship	6	6–30	25.00 (23.00, 26.50)
Family and Friends’ Recognition	7	7–35	28.00 (26.00, 31.00)
Team Belongingness	5	5–25	21.00 (19.00, 22.00)
Personal Growth	6	6–30	25.00 (23.00, 27.00)
Total Score	29	29–145	117.72 ± 12.34

### 3.4 Correlation analysis between career benefits perception and core competencies of pediatric nurse specialists

Table 4 showed a significant positive correlation between the core competencies of pediatric nurse specialists and various dimensions of career benefits perception (*p* < 0.01). Specifically, the total score of core competencies correlates with positive occupational perception, good nurse-patient relationship, family and friends' recognition, team belongingness, personal growth, and the total score of career benefits perception, with correlation coefficients of 0.803, 0.794, 0.761, 0.756, 0.535, and 0.849, respectively. In particular, clinical nursing practice ability and communication and coordination ability are highly correlated with multiple dimensions of career benefits perception.

**Table 4:** Correlation analysis between core competencies and career benefits perception of pediatric specialist nurses (*n* = 128, r).

Dimension	Basic professional qualities	Clinical nursing practice ability	Management and guidance ability	Communication and coordination ability	Professional development ability	Total core competency score
Positive Occupational Perception	0.734**	0.782**	0.778**	0.788**	0.787**	0.803**
Good Nurse-Patient Relationship	0.741**	0.772**	0.765**	0.776**	0.777**	0.794**
Family and Friends’ Recognition	0.579**	0.762**	0.754**	0.764**	0.760**	0.761**
Team Belongingness	0.572**	0.758**	0.747**	0.757**	0.755**	0.756**
Personal Growth	0.537**	0.520**	0.505**	0.513**	0.516**	0.535**
Total Career Benefits Perception Score	0.729**	0.837**	0.826**	0.838**	0.837**	0.849**

** Indicates *p* < 0.01.

### 3.5 Comparison of core competency scores of pediatric nurse specialists with different baseline characteristics

There are significant differences in the core competency scores of pediatric nurse specialists with different baseline characteristics. Specifically, years of work experience and employment type significantly impact core competency scores (*p* < 0.001). Nurses with longer work experience, higher professional titles, and more stable employment (e.g., civil service) have higher core competency scores. However, gender, age, marital status, professional title, and educational background do not significantly affect core competency scores (*p* > 0.05) (Table 5).

**Table 5:** Comparison of core competency scores of pediatric specialist nurses with different baseline characteristics.

Information		Number of cases	Score (mean ± SD)	*t/F*	*P*
Sex	Male	2	149.50 ± 9.12	1.301	0.195
	Female	126	131.96 ± 18.97		
Age (years)	21–30	83	131.65 ± 14.88	1.966	0.144
	31–40	37	129.16 ± 22.70		
	>40	8	142.75 ± 16.58		
Marital status	Unmarried	28	128.93 ± 20.73	0.434	0.649
	Married	98	132.33 ± 16.60		
	Divorced	2	135.00 ± 35.36		
Years of work (years)	≤5	28	114.07 ± 15.05	29.616	<0.001
	6–10	61	133.34 ± 13.78		
	11–20	30	136.00 ± 13.59		
	>20	9	160.00 ± 6.56		
Academic qualifications	Bachelor's Degree	109	131.47 ± 18.17	−0.239	0.811
	Master and above	19	132.53 ± 15.23		
Technical title	Nurse	76	128.53 ± 16.42	2.989	0.054
	Supervisor nurse	41	136.49 ± 17.39		
	Associate chief nurse	11	134.91 ± 23.90		
Employment form	Temporary contract	23	120.09 ± 16.93	18.311	<0.001
	Long-term contract	94	131.72 ± 15.70		
	Career	11	154.91 ± 12.59		

### 3.6 Analysis of factors influencing core competency of pediatric nurse specialists

The variables with statistically significant differences in core competency scores (years of work experience and employment type) and the total score of professional benefit perception were included in the multiple linear regression equation. The values assigned to the included variables and dummy variables are shown in Table 6. The results of the multiple linear regression analysis are shown in Table 7. The total score of professional benefit perception, civil service employment, and years of work experience are positively correlated with the core competency of pediatric nurse specialists, with the total score of professional benefit perception having the most significant impact on core competency. The model test results show F = 71.403, *p* < 0.001, and the coefficient of determination *R*^2^ = 0.769, indicating that the model can explain 76.90% of the variance in core competency.

**Table 6:** Assignment table for dummy variables of general data.

Variable	Assigned values
Years of Work Experience	6–10 years = (0,1,0,0); 11–20 years = (0,0,1,0); >20 years = (0,0,0,1); Reference: ≤5 years = (0,0,0,0)
Professional Title	Senior Nurse = (0,1,0,0); Supervisor Nurse = (0,0,1,0); Deputy Chief Nurse = (0,0,0,1); Reference: Nurse = (0,0,0,0)
Employment Type	Long-term Contract = (0,1,0); Civil Service = (0,0,1); Reference: Temporary Contract = (0,0,0)

**Table 7:** Multiple linear regression analysis of factors influencing core competency of pediatric specialist nurses.

Variable	B	SE	β	t	*P*
Total Score of Professional Benefit Perception	1.007	0.083	0.656	12.131	0.000
Employment Type (Reference: Temporary Contract)					
Civil Service	8.339	4.184	0.124	1.993	0.049
Years of Work Experience (Reference: ≤5 years)					
6–10 years	12.850	4.818	0.174	2.667	0.009
11–20 years	6.813	2.816	0.153	2.419	0.017
>20 years	6.533	2.460	0.173	2.656	0.009

## 4 Discussion

The International Council of Nurses (ICN) defines the competencies of specialist nurses as “the specific knowledge, skills, judgment, and personal qualities required to provide safe and ethically sound nursing services” (19). For healthcare managers, effectively utilizing and maximizing the core competencies of specialist nurses can improve specialist training strategies and specialist management (20, 21). Due to the particular nature of patients, pediatrics has become a high-incidence department for medical disputes. Pediatric nurse specialists experience a high workload and significant psychological pressure (22). To clarify the relationship between the core competencies of pediatric nurse specialists and their sense of professional benefit, develop targeted specialist training programs, comprehensively enhance the core competencies of pediatric nurse specialists, and ensure the quality of pediatric nursing care, a study was conducted through a questionnaire survey in Hebei Province from August to September 2023 to investigate the correlation between the core competencies of pediatric nurse specialists and their sense of professional benefit.

The results of this study show that the total core competency score of 128 pediatric nurse specialists was (132.23 ± 18.96), indicating a relatively high level. Analysis of various dimensions revealed that clinical nursing practice skills scored the highest (47.86 ± 5.93), while management and guidance skills scored relatively lower (19.08 ± 3.19). This may be related to the unique characteristics of pediatric patients. Children under the care of pediatric nurse specialists often present unique psychological and emotional needs compared to adults, requiring more attention and care. Additionally, the rapid onset, severity, and rapid changes in pediatric diseases demand more energy from pediatric nurse specialists in clinical practice. This focus on clinical practice can enhance their clinical nursing skills but may lead to neglecting the development of management and guidance skills (23–25). Table 3 shows that the professional benefit score of pediatric nurse specialists was 117.72 ± 12.34, indicating a moderately high level. This might be because pediatric nurse specialists have rich clinical practice experience, and while utilizing their specialist nursing roles and functions, they enhance their specialist capabilities and gain recognition from patients and colleagues. Additionally, as the public increasingly recognizes the importance of nursing work, pediatric nurse specialists receive more social and family support, which helps them realize their self-worth and enhances their sense of professional benefit (26, 27). However, some nurses have a relatively low level of professional benefit, which may be due to limited career development opportunities, lack of job autonomy, and a perceived low position, leading to feelings of alienation from their profession and reducing their sense of professional benefit (28, 29).

In this survey, the analysis of the correlation between the scores of each dimension and the total scores of professional benefit and core competencies for 128 pediatric nurse specialists showed that the total core competency score was positively correlated with positive professional perception, good nurse-patient relationships, recognition by friends and family, team belonging, personal growth, and the total score of professional benefit. Notably, clinical nursing practice skills and communication and coordination skills, which are the most prominent and important aspects of core competencies, showed a high correlation with multiple dimensions of professional benefit (*p* < 0.01). Additionally, the results of multiple linear regression analysis indicated that the total score of professional benefit had a significant impact on the development of core competencies (*p* < 0.001), suggesting that as pediatric nurse specialists' sense of professional benefit increases, their core competencies also improve accordingly.

In addition to professional benefit, the study also explored the relationship between general demographic information and nurses' core competencies. The results showed that pediatric nurse specialists with longer working years had significantly higher core competencies than those with shorter working years. This may be related to the accumulation of clinical experience and the increased frequency of training and assessments over time, which leads to more evident improvements in core competencies (30, 31). Moreover, pediatric nurse specialists with permanent positions (tenured staff) exhibited significantly higher core competencies compared to those with contractual positions. Multiple linear regression analysis revealed a positive correlation between having a permanent position and the core competencies of pediatric nurse specialists (*p* < 0.05). The instability of their employment relationship might influence the relatively lower core competencies of contractual nurses. Studies have indicated that many hospitals do not invest adequately in non-tenured nurses and do not prioritize their training (8, 32). Additionally, there are issues of inequality between tenured and contractual nurses, such as unequal pay for equal work, which severely affects the work motivation of contractual nurses and hinders the improvement of their core competencies. Therefore, it is essential to emphasize and encourage contractual nurses, focusing on their training and reasonably arranging their promotion paths to enhance their core competencies.

The findings reveal that management and guidance skills scored lower compared to other core competencies, which may stem from structural and educational factors inherent to pediatric nursing. Pediatric nurses often prioritize acute clinical care and communication with young patients, leaving limited opportunities to engage in managerial roles or leadership training (23). A study by Jokiniemi et al. (31) in Nordic countries similarly identified gaps in leadership competencies among specialist nurses, attributing this to a curriculum emphasis on clinical skills over administrative training (31). This aligns with our results, suggesting a global need to integrate management education into pediatric nursing programs. Additionally, cultural factors, such as hierarchical workplace dynamics in Chinese hospitals, may discourage junior nurses from taking initiative in decision-making roles, further exacerbating the deficit (32).

The strong correlation between career benefit perception and core competencies underscores the importance of fostering job satisfaction through policy interventions. For instance, contractual nurses’ lower competency scores highlight systemic inequities in training access and career advancement, a pattern also observed in gerontological nursing studies (30). To address this, hospitals could adopt standardized competency-based training frameworks, as proposed by Chen et al. (32), which have proven effective in enhancing both skills and job satisfaction among specialist nurses (8). Furthermore, comparative studies in emergency nursing demonstrate that mentorship programs and structured leadership pathways significantly improve management competencies (6), offering actionable strategies for pediatric nursing managers. By addressing these structural and educational barriers, institutions can elevate overall nursing quality, aligning with international benchmarks for specialist nurse training (19).

This study has several limitations. First, convenience sampling from three tertiary hospitals in Hebei Province limits generalizability to diverse regions or healthcare settings, compounded by a female-dominated sample. Second, the cross-sectional design precludes causal conclusions between professional benefits and core competencies, necessitating longitudinal research. Third, while validated scales were used, the core competency tool may overlook pediatric-specific skills, and self-reported benefits risk bias; integrating objective metrics would improve validity. Fourth, unmeasured organizational factors, such as hospital policies or leadership support, may confound associations. Fifth, employment type classifications lacked detail on structural disparities, obscuring competency determinants. Finally, the minimal male sample hindered gender analysis. To address these limitations, future studies should adopt multicenter randomized sampling, mixed-method approaches, and prospective cohorts.

## 5 Conclusion

Among the sampled pediatric nurse specialists in this study, core competencies were moderately high, though further development is warranted. Professional benefit was a major influencing factor. Nursing managers should enhance interventions aimed at improving the professional benefit of pediatric nurses to further stimulate the enhancement of core competencies and provide high-quality clinical nursing services for pediatric patients. Additionally, efforts should be made to strengthen the training of contractual nurses to improve overall core competencies.

## Data Availability

The original contributions presented in the study are included in the article/Supplementary Material, further inquiries can be directed to the corresponding author/s.
